# Spatiotemporal evolution of corneal biomechanical properties after cross-linking based on optical coherence elastography

**DOI:** 10.3389/fbioe.2026.1976908

**Published:** 2026-10-13

**Authors:** Yuqi Liu, Yongkang Zhao, Bi Hu, Zongzheng Zou, Haixia Zhang

**Affiliations:** 1 School of Biomedical Engineering, Capital Medical University, Beijing, China; 2 Beijing Key Laboratory of Intelligent Diagnosis Technology and Equipment for Optic Nerve-Related Eye Diseases, Beijing, China; 3 School of Mechanics and Engineering Science, Peking University, Beijing, China; 4 Beijing Institute of Ophthalmology, Beijing Tongren Eye Center, Beijing Tongren Hospital, Capital Medical University, Beijing, China; 5 Beijing Ophthalmology & Visual Sciences Key Laboratory, Beijing, China; 6 Beijing Key Laboratory of Clinical Engineering Solutions for Mental Health, Beijing, China

**Keywords:** biomechanics, cornea, corneal cross-linking, elastic modulus, optical coherence elastography

## Abstract

**Purpose:**

To characterize the spatiotemporal evolution of corneal biomechanical properties after corneal cross-linking (CXL) using optical coherence elastography (OCE), with particular emphasis on regional changes in a hemicorneal partial-CXL model and temporal responses to standard and accelerated CXL (ACXL).

**Methods:**

A silicone corneal phantom was constructed for system validation. Seven New Zealand white rabbits underwent hemicorneal partial-CXL in both eyes to investigate regional biomechanical changes. In addition, 11 rabbits received standard CXL or ACXL in contralateral eyes. OCE measurements were performed before and after treatment, and Corvis ST was additionally performed for complementary biomechanical assessment.

**Results:**

OCE identified spatial biomechanical heterogeneity and quantified regional and temporal changes after CXL. In the hemicorneal partial-CXL model, linear mixed-effects analysis demonstrated a significant region-by-time interaction (*P* < 0.001). The OCE-derived elastic modulus in the treated region was significantly higher than that in the untreated region at all postoperative time points (*P* < 0.001). Compared with baseline, the treated region increased by 38.6%, 31.1%, and 56.8% at 1 week, 2 weeks, and 1 month, respectively, while the untreated region remained stable. The elastic modulus exhibited a temporal remodeling pattern characterized by an early increase, a mild decline at the intermediate stage, and a subsequent increase. Standard CXL and ACXL showed similar time-dependent biomechanical changes, with no significant differences between protocols at corresponding postoperative time points.

**Conclusion:**

OCE revealed spatial heterogeneity and dynamic remodeling of corneal biomechanics after CXL. These findings support OCE as a tool for spatially resolved and longitudinal evaluation of CXL-induced biomechanical changes and provide a basis for future personalized CXL strategies.

## 1 Introduction

Keratoconus (KC) is a corneal ectatic disorder characterized by progressive localized corneal protrusion, with its primary pathological basis being a reduction in corneal biomechanical strength and spatial heterogeneity (Wollensak et al., 2003). Corneal cross-linking (CXL) is performed in the presence of riboflavin as a photosensitizer, where ultraviolet-A (UVA) irradiation induces the formation of additional covalent bonds between stromal collagen fibers, thereby enhancing corneal biomechanical strength and resistance to deformation (McCall et al., 2010; Yang et al., 2024). It has become the standard treatment for slowing or halting the progression of corneal ectatic diseases.

In clinical practice, CXL achieves corneal stiffening by adjusting irradiance and exposure time under a constant total energy dose, thereby inducing collagen CXL in the corneal stroma. Standard CXL and accelerated CXL (ACXL) have been developed for different clinical scenarios, with ACXL increasing irradiance while reducing exposure time to improve treatment efficiency and meet varying clinical demands (Hafezi et al., 2025; Bouheraoua et al., 2014; Zheng et al., 2023). With increasing recognition of the spatial heterogeneity of KC, personalized CXL has gained growing attention. Continuous monitoring of biomechanical remodeling in different corneal regions after CXL is essential for evaluating the efficacy of personalized treatment strategies.

At present, clinical devices such as the Ocular Response Analyzer (ORA) and Corneal Visualization Scheimpflug Technology (Corvis ST) can provide global corneal stiffness parameters but are unable to resolve local biomechanical variations (Wang et al., 2023; Liu et al., 2024). Brillouin microscopy enables high-resolution mapping of longitudinal elastic modulus; however, it cannot measure shear or tensile moduli, which are critical for describing local corneal deformation under physiological loading conditions (Shao et al., 2019; Kabakova et al., 2024).

Optical coherence elastography (OCE), as an emerging elastographic imaging technique, enables *in vivo*, non-invasive, high-resolution assessment of corneal biomechanical properties, offering a promising approach for local biomechanical evaluation. Previous OCE studies have demonstrated CXL-induced increases in corneal stiffness and have compared biomechanical outcomes among different irradiation protocols (Kirby et al., 2023; Zhou et al., 2019; Singh et al., 2016a; Singh et al., 2016b). Localized OCE measurements have also shown that biomechanical differences between treated and untreated corneal regions can be spatially resolved (Kling, 2020; Zvietcovich et al., 2020a). However, the temporal evolution of regional biomechanical heterogeneity within the same cornea remains insufficiently characterized. In particular, how the biomechanical contrast between treated and untreated regions changes during postoperative remodeling has not been systematically investigated.

Based on these considerations, the present study established a hemicorneal partial-CXL model and performed serial *in vivo* OCE measurements to characterize the spatial distribution and temporal evolution of regional corneal biomechanical changes. In parallel, the biomechanical responses to standard CXL and ACXL were compared across multiple postoperative time points. The study therefore aimed to characterize the spatiotemporal biomechanical response of the cornea after CXL and to evaluate the potential of OCE for spatially resolved assessment and temporal monitoring.

## 2 Methods

### 2.1 OCE system

This study employed an optical coherence tomography (OCT) system with a light source centered at 1,310 nm, a bandwidth of approximately 105 nm, and an A-line acquisition rate of 100 kHz, providing an axial resolution of approximately 15 μm and a lateral resolution of approximately 20 μm (Feng et al., 2022; Ramier et al., 2020). The optical power incident on the sample was maintained below 10 mW in accordance with the ANSI Z136.1–2014 safety standard. At each lateral sampling position, 1,000 repeated A-lines were acquired to form one M-scan. A total of 100 M-scans were sequentially acquired at 100 lateral positions to form a complete M-B scan, corresponding to one two-dimensional OCE dataset (Li et al., 2024). Each OCE measurement was repeated three times at the same sample location, and the resulting displacement datasets were averaged prior to subsequent processing to improve the signal-to-noise ratio.

For elastic-wave excitation, a piezoelectric transducer (PZT) was used to generate continuous sinusoidal harmonic excitation. The PZT was bonded to a wedge-shaped coupler, which was positioned in line contact with the corneal surface. The position of the excitation device was gently adjusted to ensure stable line contact and a small, consistent mechanical preload while avoiding macroscopic deformation of the cornea. The excitation frequency was selected according to the specific measurement objective. A lower frequency of 4 kHz was used for *in vivo* lateral biomechanical mapping to provide a longer propagation range across the corneal surface. For the phantom experiment, 10 kHz was selected to provide a balance between wave propagation and spatial localization of the mechanical contrast. For the depth-dependent corneal assessment, a higher frequency of 14 kHz was used to provide a shorter mechanical wavelength and greater sensitivity to localized mechanical variations. Under predefined driving amplitudes and excitation frequencies, traveling guided elastic waves with Lamb-type characteristics were generated along the corneal surface.

During data acquisition and processing, the time-dependent displacement signals of the corneal surface were first recorded. A Fourier transform was then applied along the temporal dimension to convert the displacement signals from the time domain to the frequency domain, from which the vibration amplitude and phase at the excitation frequency were extracted. Phase unwrapping was subsequently performed using the MATLAB unwrap function to eliminate discontinuities caused by phase wrapping. The relatively small lateral sampling interval helped minimize spatial phase aliasing.

To characterize elastic-wave propagation, one-dimensional displacement profiles along the corneal surface were first extracted, and a spatial Fourier transform was applied to determine the dominant wavenumber k (Li et al., 2022). The phase velocity ( c ) is calculated using Equation 1:

$$c=\frac{2\pi f}{k},$$ (1)

the OCE-derived elastic modulus (E) was estimated using the Rayleigh-wave approximation as follows:

$$E=\frac{2\rho {\left(1+\nu \right)}^{3}}{{\left(0.87+1.12\nu \right)}^{2}}c^{2},$$ (2)

where ν=0.49 is the Poisson’s ratio and $\rho =1062\;{\text{kg}/m}^{3}$ is the corneal density (Zhou et al., 2019; Kennedy et al., 2014).

Equation 2 assumes that the excited elastic wave propagates according to a Rayleigh-wave model in a semi-infinite elastic medium. Previous studies have suggested that a modified Lamb-wave model may provide a more appropriate description of elastic-wave propagation in the cornea because the corneal thickness is smaller than the characteristic wavelength (Zhou et al., 2019). Such guided-wave models can more comprehensively account for finite-thickness effects and boundary conditions and may therefore be advantageous when estimating intrinsic absolute corneal mechanical properties. Nevertheless, changes in wave velocity can still reasonably reflect relative changes in corneal stiffness, although the absolute OCE-derived elastic modulus values obtained using the Rayleigh-wave approximation may differ from those derived using more comprehensive wave-propagation models. Therefore, in the present study, the OCE-derived elastic modulus was primarily used to characterize relative spatial and temporal changes in corneal biomechanical properties. Accordingly, the absolute OCE-derived elastic modulus values reported in this study should be regarded as approximate estimates rather than intrinsic Young’s modulus values, whereas the relative spatial and temporal changes constitute the primary basis for interpretation.

For spatially resolved elastographic reconstruction, the local wave-velocity distribution was further obtained using the phase-gradient method (Li and Cao, 2017). The spatial phase gradient along the lateral direction of elastic-wave propagation was calculated, and the corresponding local wave velocity was derived from the spatial variation in phase. A finite-difference kernel was used to estimate the phase gradient, and no additional spatial smoothing or averaging was performed before elastic-modulus inversion. The local OCE-derived elastic modulus distribution was subsequently reconstructed from the local wave-velocity map using Equation 2.

### 2.2 Fabrication of silicone corneal phantoms and system validation

To evaluate the capability of the OCE system to detect spatial mechanical gradients and relative stiffness differences, a centrally softened silicone corneal phantom was fabricated. Silicone material with a viscosity of 10 Pa s (type 107) was used as the base, to which silicone oil (for modulus adjustment), 5% crosslinker (tetraethyl orthosilicate), 2% catalyst (dibutyltin dilaurate), and an appropriate amount of fumed silica were added (Tong et al., 2025). The mixture was thoroughly blended to prepare the silicone material, where a higher silicone oil content resulted in a lower modulus after curing. A silicone oil volume fraction of 33.3% was used in the central region, whereas a volume fraction of 16.7% was used in the peripheral region.

The corneal mold was designed with a diameter of 12 mm, a central thickness of 0.5 mm, a peripheral thickness of 1 mm, and an anterior surface apex curvature radius of 9.4 mm. Low-modulus silicone material was first deposited into the central region of the mold and allowed to cure. High-modulus silicone was then poured into the mold for secondary molding, yielding a corneal phantom with a softened central zone and stiffer peripheral tissue (Tong et al., 2025). The phantom was measured by OCE with an excitation frequency of 10 kHz (Figure 1a). The elastic moduli of the two silicone formulations reported in our previous study were used as reference values to evaluate whether OCE could reproduce the expected spatial stiffness contrast (Tong et al., 2025).

**FIGURE 1:**
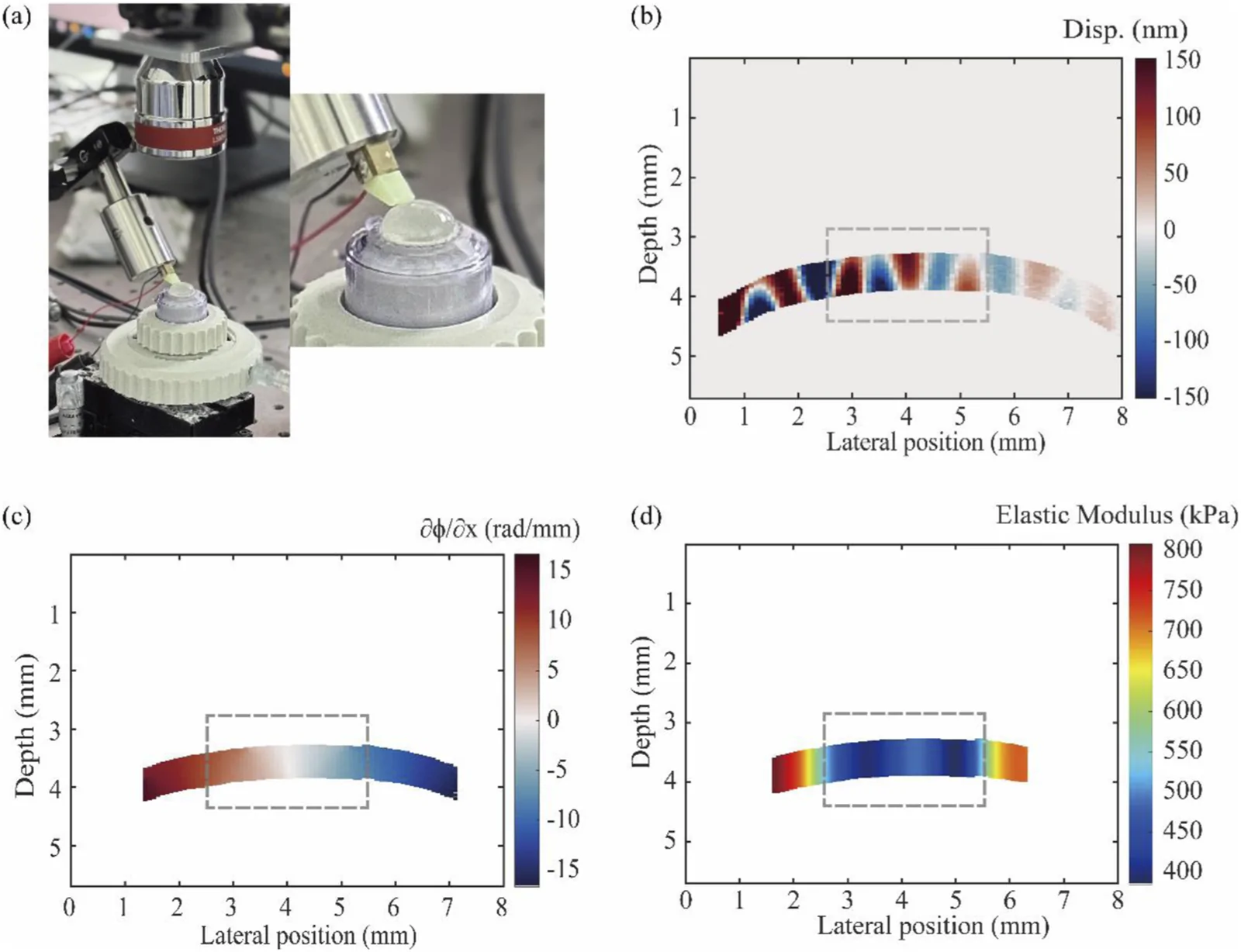
OCE assessment of central softening in silicone corneal phantom. **(a)** OCE measurement of a centrally softened silicone corneal phantom. **(b)** Wave image of elastic waves excited at 10 kHz. Color represents displacement. The dashed box indicates the softened region of the corneal phantom. **(c)** Phase-gradient map of the corneal phantom under 10 kHz excitation. **(d)** Elastic modulus distribution map reconstructed using the phase-gradient algorithm.

### 2.3 Animals experiments

This study used New Zealand white rabbits (2–3 kg, both male and female) for *in vivo* experiments. All animal procedures were conducted in accordance with the ARVO Statement for the Use of Animals in Ophthalmic and Vision Research and were approved by the Institutional Animal Care and Use Committee of Capital Medical University (approval no. AEEI-2025–878). Rabbits were anesthetized by intravenous administration of 3% sodium pentobarbital solution at a dose of 1 mL/kg during the experimental procedures. At the completion of the study, the rabbits were euthanized by intravenous administration of an overdose of 3% sodium pentobarbital solution at a dose of 3 mL/kg.

#### 2.3.1 Hemicorneal partial-CXL experiment

To investigate spatially localized biomechanical changes after CXL, a hemicorneal partial-CXL model was established. A total of 7 rabbits were enrolled, and both eyes were included (n = 14). Hemicorneal partial-CXL surgery was performed on all eyes. For each cornea, the corneal surface was divided into two halves: one half received CXL treatment, while the contralateral half served as an internal self-control. The treated side was randomly assigned to either the left or right half of the cornea. After corneal epithelium debridement, 1% riboflavin solution (Avedro, United States) was instilled to infiltrate the corneal tissue for 30 min, with 1–2 drops supplemented every 3 min. After riboflavin saturation, UVA irradiation was delivered using a UV-X TM2000 UVA irradiation system (IROC, Switzerland) at a working distance of 50 ± 5 mm, with an irradiation diameter of 8 mm and a wavelength of 365 nm. The surface irradiance was set at 3 mW/cm^2^ for 30 min and was verified using a calibrated UVA radiometer before irradiation. Riboflavin solution was supplemented every 3 min during UVA exposure.

For hemicorneal partial-CXL, the untreated half of the cornea was shielded with a metal occluder during UVA irradiation. The straight edge of the occluder was aligned through the corneal apex to divide the corneal surface into approximately equal irradiated and shielded halves, thereby providing a standardized treatment boundary. For quantitative regional analysis, the treated and untreated regions were defined strictly according to the predefined UVA irradiation boundary. All measurement locations within the irradiated half were assigned to the CXL-treated region, whereas those within the shielded, non-irradiated half were assigned to the untreated region. The same geometric region definition was applied at all postoperative follow-up time points to maintain consistency in regional comparisons.

OCE measurements at an excitation frequency of 4 kHz were carried out preoperatively for baseline data, and repeated at 1 week, 2 weeks and 1 month postoperatively to analyze the spatial distribution changes of corneal biomechanical properties (Figure 2a). To quantitatively characterize the biomechanical transition between the treated and untreated regions, lateral OCE-derived elastic modulus profiles across the predefined irradiation boundary were extracted. The biomechanical transition-zone width was defined as the lateral distance over which the elastic modulus changed from 10% to 90% between the untreated and treated region levels.

**FIGURE 2:**
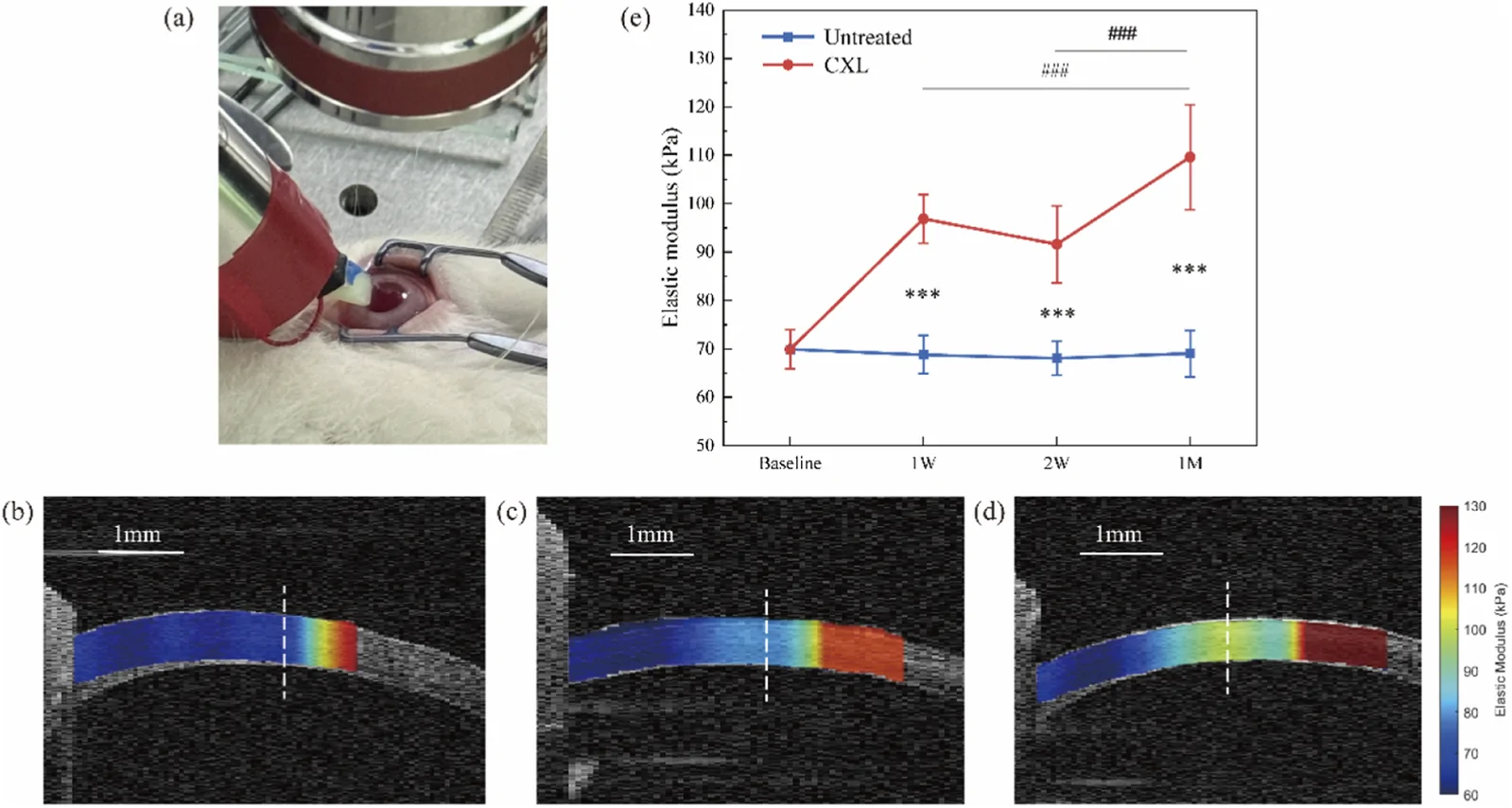
OCE measurement and biomechanical evaluation of rabbit cornea after hemicorneal partial-CXL. **(a)** Representative OCE measurement setup for *in vivo* rabbit corneal detection. **(b)** Wave velocity map at 4 kHz overlaid on OCT image 1 week after hemicorneal partial-CXL. **(c)** 2 weeks. **(d)** 1 month. **(e)** Quantitative comparison of corneal elastic modulus in crosslinked and non-crosslinked regions at different time points after hemicorneal partial-CXL. ***P < 0.001, CXL vs. Untreated at the corresponding postoperative time point; ###*P* < 0.001, temporal comparisons within the CXL group.

#### 2.3.2 Standard CXL and ACXL

To investigate the effects of different CXL protocols on corneal biomechanical properties, a total of 11 rabbits were included. One eye of each rabbit received full-field standard CXL treatment (3 mW/cm^2^, 30 min), while the contralateral eye underwent ACXL treatment (9 mW/cm^2^, 10 min), with left and right eyes randomly grouped. *In vivo* OCE measurements were performed preoperatively and at 1 week, 2 weeks, and 1 month postoperatively, using an excitation frequency of 4 kHz. Central corneal thickness (CCT) was obtained from the structural OCT images acquired by the OCE system at each measurement time point in both the standard CXL and ACXL groups.

In addition, one rabbit eye treated with standard full-field CXL was selected at 1 month postoperatively for an additional descriptive assessment of depth-dependent corneal biomechanics. OCE measurements were conducted at a higher excitation frequency of 14 kHz to characterize the depth-dependent distribution of corneal mechanical properties in this specimen.

#### 2.3.3 Corvis ST measurements

For complementary biomechanical assessment, three rabbits that underwent full-field standard CXL were randomly selected. *In vivo* measurements were performed using Corvis ST before surgery and at 1 month postoperatively to record corneal dynamic parameters, including central corneal thickness (CCT), corneal stiffness parameter (SP-A1), deformation amplitude (DA), first and second applanation times (A1 Time and A2 Time), highest concavity time (HCT), peak distance (PD), and radius at the highest concavity state (R). Based on Reissner’s theory, these parameters were used to estimate the Corvis ST-derived elastic modulus, providing an additional biomechanical measure for evaluating CXL-related changes and for comparison with the OCE findings (Qin et al., 2019).

### 2.4 Data analysis

All data processing and statistical analyses were performed using MATLAB (MathWorks, Natick, MA, United States) and SPSS software (IBM Corp., Armonk, NY, United States). Data are presented as mean ± standard deviation (SD). For the hemicorneal partial-CXL experiment, region, postoperative time, and their interaction were included as fixed effects in the linear mixed-effects model. For the standard CXL/ACXL experiment, treatment, time, and their interaction were included as fixed effects. In both models, random intercepts were included for rabbit and for eye nested within rabbit to account for correlation between fellow eyes from the same animal and repeated measurements within the same eye. A variance-components structure was used for the random effects, with independent residual errors of homogeneous variance. Models were fitted using all available observations, and missing measurements were not imputed. Post hoc pairwise comparisons were adjusted using the Bonferroni method. A two-sided *P* < 0.05 was considered statistically significant. Given the limited number of Corvis ST measurements, these data were summarized descriptively using mean ± SD and the observed paired changes.

## 3 Results

### 3.1 Central softening in silicone corneal phantom

To validate the capability of OCE for detecting spatial mechanical heterogeneity, a silicone corneal phantom containing regions with different mechanical properties was constructed. Based on our previous characterization of silicone mixtures, the peripheral and central regions were designed using materials with silicone oil volume fractions of 16.7% and 33.3%, respectively, which exhibited a known elastic modulus ratio of approximately 2.16 (Tong et al., 2025).

In OCE measurements of the phantom, the excitation frequency was set to 10 kHz. The surface displacement field of the phantom is shown in Figure 1b. The acquired phase information was first phase-unwrapped to remove discontinuities caused by phase wrapping. The spatial phase-gradient distribution was then calculated (Figure 1c) to characterize the local spatial rate of phase variation associated with wave propagation. Based on the phase-gradient information, the corresponding wave velocity distribution was further derived. Finally, the OCE-derived elastic modulus map of the corneal phantom was reconstructed from the velocity distribution, enabling quantitative characterization of its local mechanical heterogeneity. The results showed that the OCE-derived modulus in the peripheral region was approximately twice that of the central region (Figure 1d). Although discrepancies in absolute modulus values were observed between the OCE-derived estimates and uniaxial tensile testing due to differences in loading conditions and measurement scales, the modulus ratio between the two regions remained highly consistent. These findings indicate that the OCE system can effectively reflect the relative spatial distribution of mechanical properties within the corneal phantom.

### 3.2 Corneal mechanical changes after CXL

In vivo OCE measurements of rabbit eyes, an excitation frequency of 4 kHz was used for evaluating the distribution of corneal lateral elastic modulus. Figures 2b–d show the corneal lateral OCE-derived elastic modulus maps reconstructed using the phase-gradient algorithm at 1 week, 2 weeks, and 1 month after hemicorneal partial-CXL, respectively. The dashed line indicates the predefined irradiation boundary during CXL. Clear biomechanical differences between the untreated and treated regions were observed at all three postoperative time points. Rather than an abrupt change at the irradiation boundary, the modulus exhibited a continuous spatial transition between the two regions. The OCE-defined biomechanical transition-zone widths in the representative maps were approximately 0.49 mm at 1 week, 1.25 mm at 2 weeks, and 1.71 mm at 1 month. The transition zone therefore showed a progressive broadening over the postoperative observation period.

To further quantify differences in elastic modulus, we performed separate calculations for the crosslinked and non-crosslinked regions within the same cornea. Figure 2e presents the quantitative analysis of temporal changes in elastic modulus in the CXL-treated and untreated regions of hemicorneal partial-CXL corneas. Mixed-effects analysis revealed a significant region-by-time interaction (*F* = 10.86, *P* < 0.001), indicating different temporal biomechanical responses between the CXL-treated and untreated regions. At 1 week, 2 weeks, and 1 month postoperatively, the elastic modulus in the CXL-treated region was significantly higher than that in the untreated region (*P* < 0.001). Compared with baseline, the elastic modulus in the CXL-treated region increased by approximately 38.6%, 31.1%, and 56.8% at 1 week, 2 weeks, and 1 month, respectively, with significant increases at all three postoperative time points (*P* < 0.001). In contrast, no significant changes from baseline were observed in the untreated region (*P* > 0.05). Within the CXL-treated region, the elastic modulus showed a modest decrease from 1 week to 2 weeks. At 1 month, the modulus was significantly higher than that at both 1 week and 2 weeks (*P* < 0.001). No significant temporal differences were observed within the untreated region.

### 3.3 Temporal changes in corneal mechanical properties under different CXL protocols

To further investigate the effects of different CXL protocols on corneal biomechanics, OCE was used to compare the biomechanical outcomes of full-field standard CXL and ACXL and to characterize the temporal changes in OCE-derived elastic modulus within each treatment protocol. Statistical analysis showed that both the standard CXL and ACXL protocols exhibited significant time-dependent changes in corneal elastic modulus (Figures 3a,b). Compared with the preoperative baseline, the elastic modulus increased significantly at 1 week, 2 weeks, and 1 month postoperatively in both treatment protocols. In each protocol, the elastic modulus showed a slight decrease at 2 weeks compared with 1 week, followed by a further increase at 1 month. This temporal pattern was consistent with the findings from the hemicorneal partial-CXL model described in Section 3.2. Although the standard CXL and ACXL protocols showed similar temporal patterns in OCE-derived elastic modulus, no statistically significant between-protocol difference was detected at the corresponding postoperative time points under the present experimental conditions (Figure 3c). CCT was also evaluated in both the standard CXL and ACXL groups (Table 1). The results showed that CCT exhibited significant time-dependent changes after CXL (time effect, *P* = 0.0019). CCT significantly increased at 1 week postoperatively and subsequently decreased gradually at 2 weeks and 1 month after surgery. No significant difference in CCT was observed between the standard CXL and ACXL groups. These findings indicate that standard CXL and ACXL induced comparable temporal changes in corneal thickness.

**FIGURE 3:**
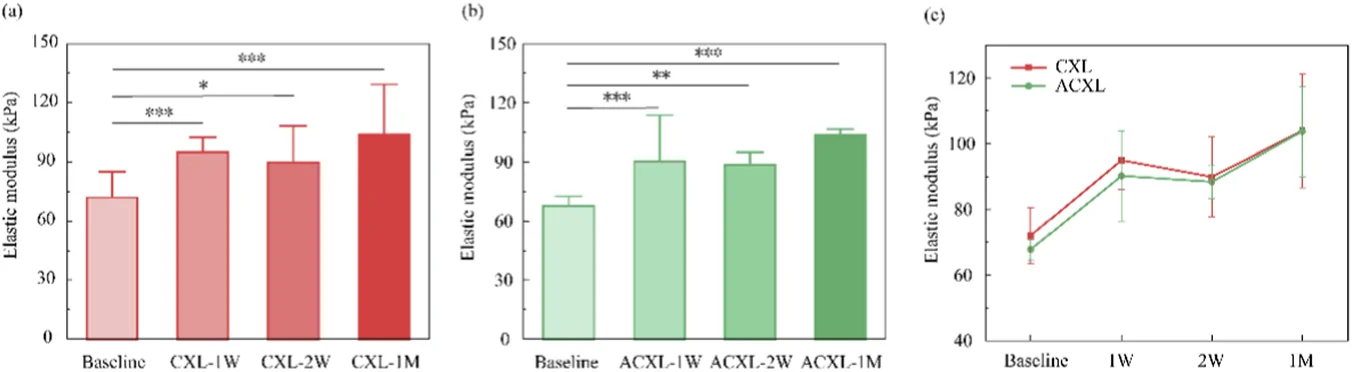
Changes in corneal elastic modulus under different CXL protocols (CXL and ACXL) at baseline, 1 week, 2 weeks, and 1 month post-surgery. **(a)** Corneal elastic modulus changes in the CXL group at baseline, 1 week, 2 weeks, and 1 month. **(b)** Corneal elastic modulus changes in the ACXL group at baseline, 1 week, 2 weeks, and 1 month. **(c)** Comparison of temporal trends in corneal elastic modulus between the CXL and ACXL groups. *P < 0.05, **P < 0.01, ***P < 0.001.

**TABLE 1:** Central corneal thickness after standard CXL and ACXL.

Treatments	Standard CXL (μm)	ACXL (μm)	*P*
Baseline	364.4 ± 33.1	361.2 ± 17.1	0.733
1 week	390.7 ± 19.2	384.8 ± 16.2	0.855
2 weeks	367.1 ± 29.5	362.0 ± 19.9	0.884
1 month	358.8 ± 22.3	353.9 ± 18.4	0.921

To characterize the depth-dependent mechanical distribution after CXL, one rabbit cornea at 1 month after full-field standard CXL was assessed using *in vivo* OCE at an excitation frequency of 14 kHz. The depth-dependent OCE-derived elastic modulus distribution was reconstructed using the phase-gradient algorithm. In this single-cornea assessment, the OCE-derived elastic modulus was higher in the anterior cornea than in the posterior cornea, demonstrating a depth-dependent mechanical gradient in this specimen. A distinct demarcation line was also visible in the OCT image near the transition region of the modulus distribution (Figure 4). Although this observation was obtained from a single cornea, it provides a descriptive example of depth-dependent biomechanical heterogeneity after CXL and is consistent with previously reported anterior–posterior mechanical differences.

**FIGURE 4:**
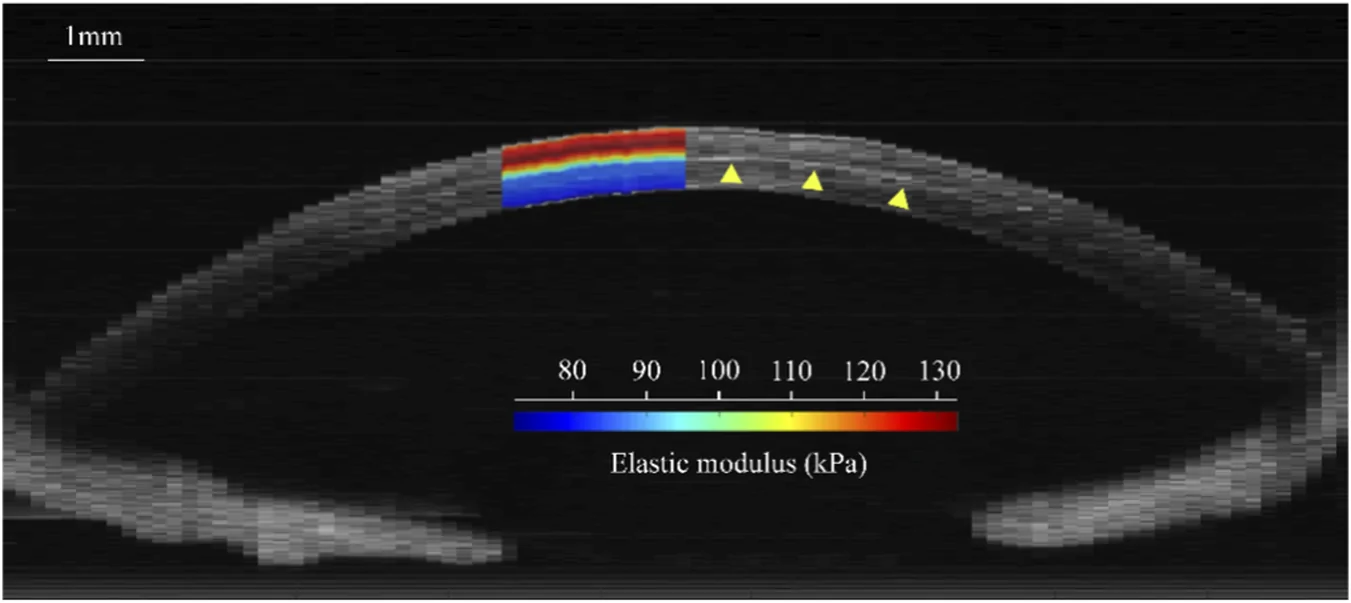
Depth-resolved OCE imaging of the cornea 1 month after full-field standard CXL. The yellow triangles indicate the CXL line.

### 3.4 Corvis measurement results

Figure 5 presents the paired Corvis ST measurements obtained before and 1 month after full-field standard CXL in three rabbits. Because this assessment involved a small subset of animals, the results were interpreted descriptively, with emphasis on the direction and magnitude of the observed changes. At 1 month after CXL, SP-A1 and A1 time showed increasing trends, whereas CCT, DA, A2 time, and R showed decreasing trends. The Corvis ST-derived elastic modulus increased from 491.97 ± 84.03 kPa at baseline to 762.93 ± 29.47 kPa at 1 month, corresponding to an approximately 55.1% increase. Overall, these directional changes were consistent with increased corneal stiffness after CXL and showed a biomechanical trend similar to that observed by OCE.

**FIGURE 5:**
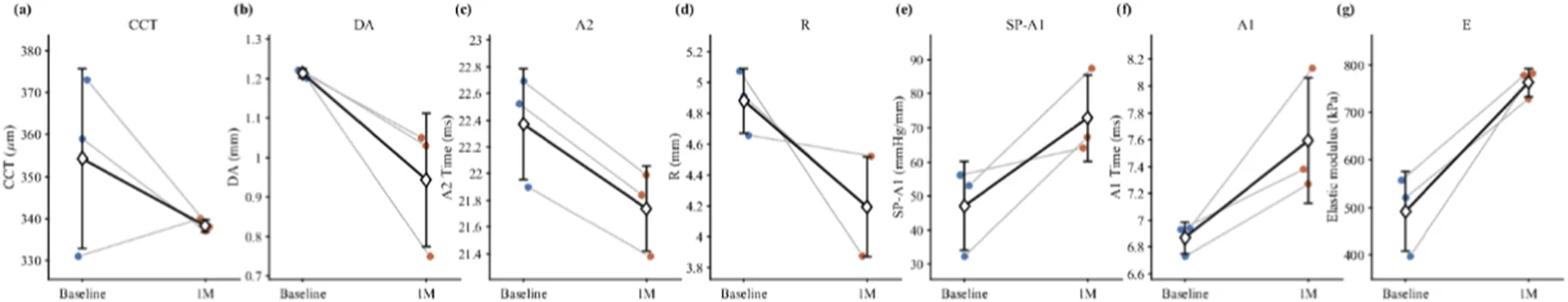
Changes in corneal biomechanical parameters before and after CXL. **(a)** Central corneal thickness (CCT); **(b)** deformation amplitude (DA); **(c)** second applanation time (A2 Time); **(d)** radius at the highest concavity state (R); **(e)** corneal stiffness parameter at first applanation (SP-A1); **(f)** first applanation time (A1 Time); and **(g)** Corvis ST-derived elastic modulus (E). Blue and orange indicate preoperative and postoperative data, respectively.

Notably, the reduction in CCT after CXL is consistent with previously reported corneal stromal compaction and dehydration effects associated with the cross-linking process. Therefore, changes in corneal thickness should be carefully considered when interpreting stiffness-related parameters, as variations in CCT may influence biomechanical response measurements and potentially affect direct comparisons of absolute elastic modulus values.

## 4 Discussion

In this study, OCE was applied to evaluate corneal biomechanical remodeling after different CXL protocols *in vivo*. Compared with conventional methods that provide only global biomechanical information, OCE provides spatially resolved OCE-derived elastic modulus maps, enabling comprehensive assessment of lateral distribution, depth-dependent gradients, and temporal changes after CXL. Therefore, OCE may provide a useful tool for spatially resolved assessment and temporal monitoring after CXL and provides preclinical biomechanical information relevant to future investigations of spatially customized CXL strategies.

Different excitation frequencies were used for different experimental objectives in this study. Lower-frequency excitation (4 kHz) was applied for lateral biomechanical mapping because it provides longer wavelength propagation and a larger effective detection range, whereas higher-frequency excitation (14 kHz) was used for depth-dependent analysis because shorter wavelengths improve sensitivity to localized mechanical variations. The 10 kHz excitation used in phantom validation represented a balance between spatial sensitivity and stable wave propagation. Therefore, frequency selection was optimized according to the specific measurement objective rather than representing a methodological inconsistency.

Before application in animal models, this study first evaluated the ability of OCE to quantitatively characterize spatial mechanical differences using a silicone corneal phantom. Although discrepancies existed between OCE-derived elastic modulus values and conventional mechanical testing in terms of absolute elastic modulus values, the relative trends of mechanical variation were highly consistent, indicating that OCE is suitable for evaluating internal mechanical heterogeneity and regional mechanical differences within tissues. A transition zone between the central softened region and peripheral stiff region was observed in the phantom measurements. This finding may be attributed to the fabrication process, as secondary molding can introduce interfacial mixing or overlapping between silicone materials with different mechanical properties, preventing the formation of an infinitely sharp boundary. Importantly, the presence of this transition region demonstrates that OCE is sensitive not only to regional stiffness differences but also to spatial variations in mechanical properties.

In the hemicorneal partial-CXL model, OCE clearly distinguished the lateral mechanical properties of the treated and untreated regions and revealed a finite biomechanical transition near the predefined irradiation boundary (Figure 2). Previous localized CXL studies using Brillouin microscopy similarly demonstrated that a sharply defined irradiation boundary does not produce an equally sharp biomechanical boundary, with a transition zone reported between fully crosslinked and non-crosslinked corneal regions (Webb et al., 2019). The progressive broadening observed during the first postoperative month may reflect the dynamic stromal response following CXL. Previous rabbit studies have demonstrated ongoing keratocyte repopulation and remodeling during this period, with activated keratocytes particularly evident near the periphery of the irradiated region at approximately 4–6 weeks (Wollensak et al., 2007). Other longitudinal rabbit studies have shown that stromal changes continue to evolve during the first month, with spatial extension of the healing response and cellular migration at the margins of the CXL region (Bradford et al., 2018). These processes may contribute to the evolving biomechanical transition between treated and untreated tissue.

Beyond lateral mechanical distribution, the single-cornea depth-dependent assessment showed a higher OCE-derived elastic modulus in the anterior cornea than in the posterior cornea at 1 month after CXL. This observation provides a descriptive example of depth-dependent biomechanical heterogeneity after CXL and is consistent with previous Brillouin microscopy and OCE studies reporting more pronounced biomechanical changes in the anterior stroma after CXL, with relatively limited changes in the posterior stroma (Scarcelli et al., 2013). Such depth-dependent differences may be related to riboflavin-mediated UVA absorption and the limited penetration depth of the crosslinking reaction (Regnault et al., 2023; Mazzotta et al., 2014). Although based on a single cornea, the observed anterior–posterior pattern is biologically consistent with previous reports, larger studies are needed to further establish its reproducibility and temporal characteristics. The higher excitation frequency provides a shorter mechanical wavelength and may increase sensitivity to localized mechanical variations, whereas depth information is obtained from the depth-resolved OCT measurements. Therefore, the excitation frequency may be selected according to the measurement objective, balancing wave propagation range and sensitivity to localized biomechanical variations.

This study further demonstrated that corneal biomechanical changes after CXL exhibit clear temporal dependence. The early increase in OCE-derived elastic modulus may be associated with the immediate biomechanical effects of photochemical collagen crosslinking, whereas the modest decrease from 1 to 2 weeks followed by a further increase at 1 month may reflect dynamic changes during postoperative stromal remodeling (Randleman et al., 2015; Yang et al., 2023). These temporal biomechanical changes are consistent with the dynamic process of CXL-induced stromal modification reported in previous studies. Further integration of OCE with structural imaging approaches, such as histological analysis or second harmonic generation imaging, may provide additional insights into the relationship between biomechanical remodeling and collagen structural evolution after CXL. SHG imaging studies have also shown that collagen fiber organization continues to improve over an extended period after CXL (Germann et al., 2020).

In this study, standard CXL and ACXL exhibited similar temporal biomechanical patterns during the 1-month follow-up. However, no significant between-protocol difference was detected under the present experimental conditions. However, the biomechanical efficacy of CXL is influenced not only by total energy but also by oxygen diffusion, riboflavin distribution, and tissue structure. Therefore, the long-term biomechanical outcomes of different protocols require further investigation (Dina et al., 2025; Khairy et al., 2019).

Recent clinical studies have also explored modified accelerated CXL strategies for corneas that may not be suitable for conventional protocols. Argon and Avcı reported contact lens-assisted accelerated CXL using a Nelfilcon A hydrogel lens in progressive keratoconus with thin corneas (post-debridement thickness ≤ 400 μm), demonstrating improvement in visual acuity, modest keratometric flattening, and preservation of endothelial cell density during 6 months of follow-up (Argon and Avcı, 2026). These findings illustrate the increasing adaptation of CXL protocols to individual corneal characteristics and further highlight the potential value of spatially resolved biomechanical assessment for evaluating treatment responses across different CXL strategies.

From the perspective of relative biomechanical change, the magnitude of CXL-induced stiffening observed in this study was consistent with previous *in vivo* OCE reports. Previous studies reported an approximately 52.5% increase in Young’s modulus after CXL in rabbit corneas (Zhou et al., 2019), which is comparable to the relative changes observed in the present OCE measurements. The Corvis ST-derived elastic modulus also showed a descriptive increase of approximately 55.1% at 1 month, with a direction of change consistent with the OCE findings. These observations indicate broadly similar biomechanical trends across the two measurement approaches, while formal comparison between them will require larger datasets.

While these findings highlight the potential value of spatially resolved biomechanical assessment, the present results were obtained in rabbit corneas with a postoperative follow-up of up to 1 month and should therefore be considered within a preclinical context. Differences in corneal geometry, thickness, wound-healing responses, and longer-term biomechanical remodeling between rabbit and human corneas may influence the spatial and temporal patterns observed after CXL. Further studies in human corneas with longer follow-up will be important for evaluating the clinical applicability of OCE-based spatial biomechanical assessment and its potential role in spatially customized CXL.

This study has several limitations. First, in lateral mechanical imaging after hemicorneal partial-CXL, the use of low-frequency excitation expanded the scanning range but still resulted in signal attenuation in peripheral regions. Future studies may improve measurement completeness by optimizing excitation strategies, adopting multi-point acquisition approaches, and enhancing signal-to-noise performance. Second, the depth-dependent biomechanical analysis was based on a single cornea and is therefore primarily interpreted as a descriptive observation. Nevertheless, the observed anterior–posterior mechanical pattern was consistent with previous reports. Further studies with larger cohorts and additional postoperative time points are needed to confirm the reproducibility and temporal characteristics of this pattern. Another methodological consideration is the use of the Rayleigh-wave approximation for modulus estimation. Because the cornea is a thin, bounded structure, this approximation does not fully account for finite-thickness effects and guided-wave behavior. Therefore, the absolute OCE-derived modulus values reported in this study should be interpreted as approximate estimates rather than intrinsic material constants. Nevertheless, because all measurements were analyzed using the same framework, the relative spatial and temporal changes constitute the primary basis for the comparisons and conclusions of this study. Future studies incorporating Lamb-wave or other guided-wave models may provide a more comprehensive characterization of absolute corneal biomechanical properties. In addition, intraocular pressure (IOP) represents an important physiological loading condition for the cornea and may influence elastic-wave propagation through changes in corneal prestress (Zvietcovich et al., 2020b). In the present study, IOP was not measured concurrently with OCE. However, in the hemicorneal partial-CXL experiment, comparisons between the crosslinked and non-crosslinked regions were performed within the same eye at the same measurement session, such that both regions were exposed to the same global IOP condition. Thus, the regional biomechanical differences observed within individual eyes are unlikely to be explained by IOP variation alone. Variations in IOP between postoperative measurement time points may have contributed to some of the observed temporal variations in OCE-derived elastic modulus. Future studies incorporating concurrent IOP measurements may further characterize the influence of physiological loading on the temporal biomechanical response after CXL. Finally, Corvis ST measurements were obtained from only three rabbits and were therefore interpreted descriptively. Although several Corvis ST parameters and the Corvis ST-derived elastic modulus showed directional changes broadly consistent with the OCE findings, the small sample size limits formal comparison between the two measurement approaches. Future studies with larger, prospectively designed cohorts are warranted to further evaluate the relationship between Corvis ST-derived and OCE-derived biomechanical measurements.

Artificial intelligence methods may further enhance OCE data analysis in future studies, including image denoising, motion correction, automatic parameter extraction, and postoperative biomechanical classification, thereby improving the efficiency of personalized CXL evaluation.

In conclusion, this study demonstrates that OCE enables spatially resolved characterization and temporal monitoring of corneal biomechanical changes after CXL, including regional differences, depth-dependent gradients, and time-dependent evolution. Serial OCE measurements revealed dynamic biomechanical responses during the early postoperative period and showed similar short-term biomechanical trajectories following standard CXL and ACXL. These findings support the potential of OCE for postoperative biomechanical monitoring and provide a basis for future investigations of spatially customized CXL strategies.

## Data Availability

The raw data supporting the conclusions of this article will be made available by the authors, without undue reservation.
